# Sclerosing Sialadenitis Is Associated With Salivary Gland Hypofunction and a Unique Gene Expression Profile in Sjögren’s Syndrome

**DOI:** 10.3389/fimmu.2021.699722

**Published:** 2021-07-30

**Authors:** Hongen Yin, Thomas J. F. Pranzatelli, Benjamin N. French, Nan Zhang, Blake M. Warner, John A. Chiorini, NIDCD/NIDCR Genomics and Computational Biology Core

**Affiliations:** ^1^Adeno-Associated Virus (AAV) Biology Section, National Institute of Dental and Craniofacial Research, National Institutes of Health, Bethesda, MD, United States; ^2^Salivary Disorders Unit, National Institute of Dental and Craniofacial Research, National Institutes of Health, Bethesda, MD, United States; ^3^Genomics and Computational Biology Core, National Institute on Deafness and Other Communication Disorders/National Institute of Dental and Craniofacial Research, Bethesda, MD, United States

**Keywords:** Sjögren’s syndrome, salivary gland interstitial fibrosis, sclerosing sialadenitis, salivary gland hypofunction, transcriptomic gene expression profile

## Abstract

**Purpose:**

To develop a novel method to quantify the amount of fibrosis in the salivary gland and to investigate the relationship between fibrosis and specific symptoms associated with Sjögren’s syndrome (SS) using this method.

**Materials and Methods:**

Paraffin-embedded labial salivary gland (LSG) slides from 20 female SS patients and their clinical and LSG pathology data were obtained from the Sjögren’s International Collaborative Clinical Alliance. Relative interstitial fibrosis area (RIFA) in Masson’s trichrome-stained LSG sections was quantified from digitally scanned slides and used for correlation analysis. Gene expression levels were assessed by microarray analysis. Core promoter accessibility for RIFA-correlated genes was determined using DNase I hypersensitive sites sequencing analysis.

**Results:**

RIFA was significantly correlated with unstimulated whole saliva flow rate in SS patients. Sixteen genes were significantly and positively correlated with RIFA. In a separate analysis, a group of differentially expressed genes was identified by comparing severe and moderate fibrosis groups. This combined set of genes was distinct from differentially expressed genes identified in lung epithelium from idiopathic pulmonary fibrosis patients compared with controls. Single-cell RNA sequencing analysis of salivary glands suggested most of the RIFA-correlated genes are expressed by fibroblasts in the gland and are in a permissive chromatin state.

**Conclusion:**

RIFA quantification is a novel method for assessing interstitial fibrosis and the impact of fibrosis on SS symptoms. Loss of gland function may be associated with salivary gland fibrosis, which is likely to be driven by a unique set of genes that are mainly expressed by fibroblasts.

## Introduction

Salivary and lacrimal gland hypofunction are hallmarks of Sjögren’s syndrome (SS) and are associated with a decreased quality of life (1). Although recovery of salivation is an essential target in therapeutic interventions for SS, the clinical pathological mechanism that causes this gland hypofunction remains unclear. Therefore, it is important to determine the underlying pathogenesis of gland hypofunction in SS.

Pathological changes in the labial salivary gland (LSG) are representative of the pathological changes in major salivary glands, such as the parotid gland (2). Therefore, LSG biopsy is a standard diagnostic method for SS, with high sensitivity and specificity, especially in patients with glandular hypofunction and negative antibodies (3). Daniels et al. identified lymphocytic infiltration and interstitial fibrosis as two characteristic microscopic changes in LSG biopsy in SS (4). Although the salivary gland focus score (FS), which represents the amount of lymphocytic infiltration, is a vital diagnostic criterion for SS and strongly suggests the presence of autoimmunity, it shows no significant contribution to salivary gland hypofunction in a large cohort clinical study (4). Therefore, it is important to understand if other pathological changes such as interstitial fibrosis is associated with salivary gland hypofunction in SS. The Sjögren’s International Collaborative Clinical Alliance (SICCA) (https://websites.ucsf.edu/content/sjogrens-international-collaborative-clinical-alliance-sicca). has established a classification for LSG microscopic characteristics. Four LSG microscopic groups were proposed specifically to assess the degree of interstitial fibrosis and lymphocytic infiltration in SS in our study. These include the following: non-specific chronic sialadenitis (NSCS; presence of scattered or focal infiltrates of lymphocytes with moderate interstitial fibrosis and gland atrophy), FLS (presence of one or more focal lymphocytic foci as the most prominent feature), sclerosing chronic sialadenitis (SCS; predominant changes are interstitial fibrosis and acinar atrophy), and FLS/SCS (combination of FLS and SCS features).

Interstitial fibrosis in epithelial organs such as the lung, liver, and kidney is commonly seen in systemic rheumatic diseases for example rheumatoid arthritis, systemic lupus erythematosus, systemic sclerosis, IgG4 disease, SS, and primary biliary cirrhosis (5) [for review, see (6–8)]. Epithelial interstitial fibrosis leads to organ failure and is frequently associated with a poor prognosis and increased mortality (9). Previously, the assessment of salivary gland fibrosis was based on either semi-quantification (10) or the microscopic classification characterized by SICCA (4). Recently, Leehan et al. reported on an objective measurement to quantify the degree of LSG fibrosis by reconstructing each hematoxylin and eosin-stained LSG section into small grids digitally (“positive” means containing ≥ 50% fibrotic tissue) and assessing the relative size of areas with fibrosis. Their result was comparable to pathologist-determined scoring (11). An additional study reported an association between the degree of salivary gland fibrosis and decreased stimulated saliva flow rate (10), suggesting that interstitial fibrosis area plays an important role in the salivary gland hypofunction in SS.

The development of sclerosing sialadenitis, in which interstitial fibrosis and subsequent gland atrophy are major histological changes, can be triggered by salivary gland epithelial cell-centered autoimmune epithelitis (1, 12). Previous studies have suggested that chronic inflammation initiates type II epithelial-mesenchymal transition (EMT) in different epithelia, such as in the kidney, liver, lung, and intestine (13, 14) [for review, see (15, 16)]. A variety of signals from immune cells and fibroblasts leads to an overproduction of collagens, laminins, elastin, and tenacins in the extracellular matrix, resulting in the formation of interstitial fibrosis. This results in replacement of epithelial cells and loss of organ function (13). In SS, a correlation between the presence of fibrosis and lymphocytic infiltration was found in several clinical studies (10, 11). It is possible that autoimmune epithelitis and other environmental changes induce EMT, promoting interstitial fibrosis.

EMT is characterized by the presence of epithelial cells that continue to exhibit epithelial-specific markers, such as epithelial cadherin (cadherin-1), and show concomitant expression of mesenchymal markers, such as fibroblast-specific protein 1, α-smooth muscle actin and cadherin-11 (17) [for review, see (16)]. Triggered by epithelial environmental changes, EMT-associated genes, such as *TGFB1* and *BMP7*, as well as the *WNT* gene family, initiate the cellular signaling circuitry that regulates EMT (13, 14) [for a review, see (15, 16)]. Expression of these genes is thought to decrease the expression of markers associated with the epithelial state and to increase the expression of markers associated with the fibroblastic state. This transition is likely to be related to epigenetic regulation, such as open chromatin accessibility [for review, see (18)].

In this study, we established a novel quantification measurement, termed relative interstitial fibrosis area (RIFA), to quantify the degree of interstitial fibrosis in LSGs and used this method to investigate the relationship of local fibrosis with gland function, as well as with transcriptomic changes in the salivary gland. Our findings suggest that salivary gland fibrosis is associated with the loss of gland function and that the increase in this type of fibrosis may be driven by a unique set of environmental factors compared with those associated with lung fibrosis.

## Materials & Methods

### Study Design

**Figure 1:**
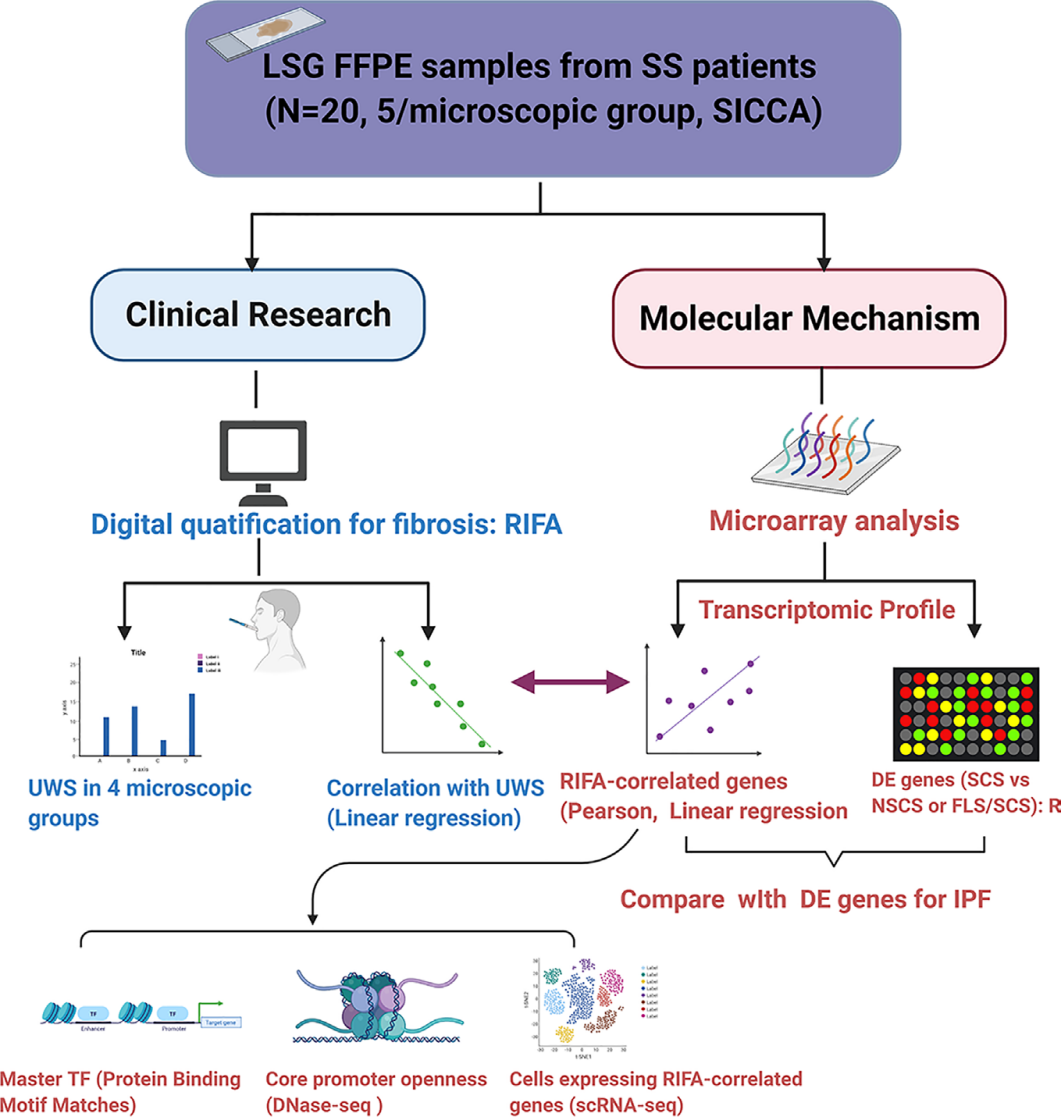
Flow chart of study design. The purpose of this study was to investigate the application of quantification of digitally scanned slides of salivary gland interstitial fibrosis and its correlation with patients’ clinical data and transcriptome changes. We developed a novel quantification method based on measuring RIFA and compared this with the conventional interstitial fibrosis evaluation methodology by microscopic classification (4). Following validation, this method was applied to study the correlation of RIFA with Sjögren’s syndrome (SS) clinical features, such as unstimulated whole saliva flow rate. Genes associated with interstitial fibrosis development could be identified by correlating RIFA with gene expression data. These genes were then compared with the list of differentially expressed genes associated with severe fibrosis using microarray data from the SCS group (severe fibrosis) versus the NSCS group or FLS/SCS group (moderate fibrosis). This transcriptome profile that was associated with sclerosing sialadenitis in SS (RIFA-correlated genes and differentially expressed genes from SCS vs. NSCS or FLS/SCS groups) was compared with differentially expressed genes in IPF. Change in expression in this group of genes was further analyzed for common transcriptional factors binding motifs, cellular source of expression, and core promoter openness.

The study design is illustrated in **Figure 1**.

### Patient Selection

The LSG samples of 20 female SS patients were selected from SICCA specimen repository (4, 19). All patients fulfilled the 2016 American College of Rheumatology/European League Against Rheumatism classification criteria for SS (20) and their clinical manifestations are summarized in **Supplementary Table 1**. Microscopic LSG changes from the selected patients were evaluated by oral pathologists from SICCA and were categorized as FLS, NSCS, FLS/SCS or SCS (4). N=5/microscopic group (N=20 total) patients were randomly selected in this study. All other clinical data, including unstimulated whole saliva (UWS) flow rate, focus score (FS), and presence of autoantibodies, such as antinuclear antibody (ANA), anti-Ro antibody (SSA) and anti-La antibody (SSB), were reported by SICCA.

### Masson’s Trichrome Staining and Interstitial Fibrosis Quantification

Formalin-fixed, paraffin-embedded LSG slides from SS patients were obtained from the SICCA International Sjögren’s Syndrome Biorepository and Data Registry (4). Masson’s trichrome staining was performed by Histoserv (Frederick, MD, USA), according to a previously published protocol (www.ihcworld.com/_protocols/special_stains/masson_trichrome.htm). Areas of interstitial fibrosis in the Masson’s trichrome–stained slides were imaged and assessed using the Aperio Scan Scope imaging technology (Leica Biosystems, Buffalo Grove, IL, USA) (21).

**Figure 2:**
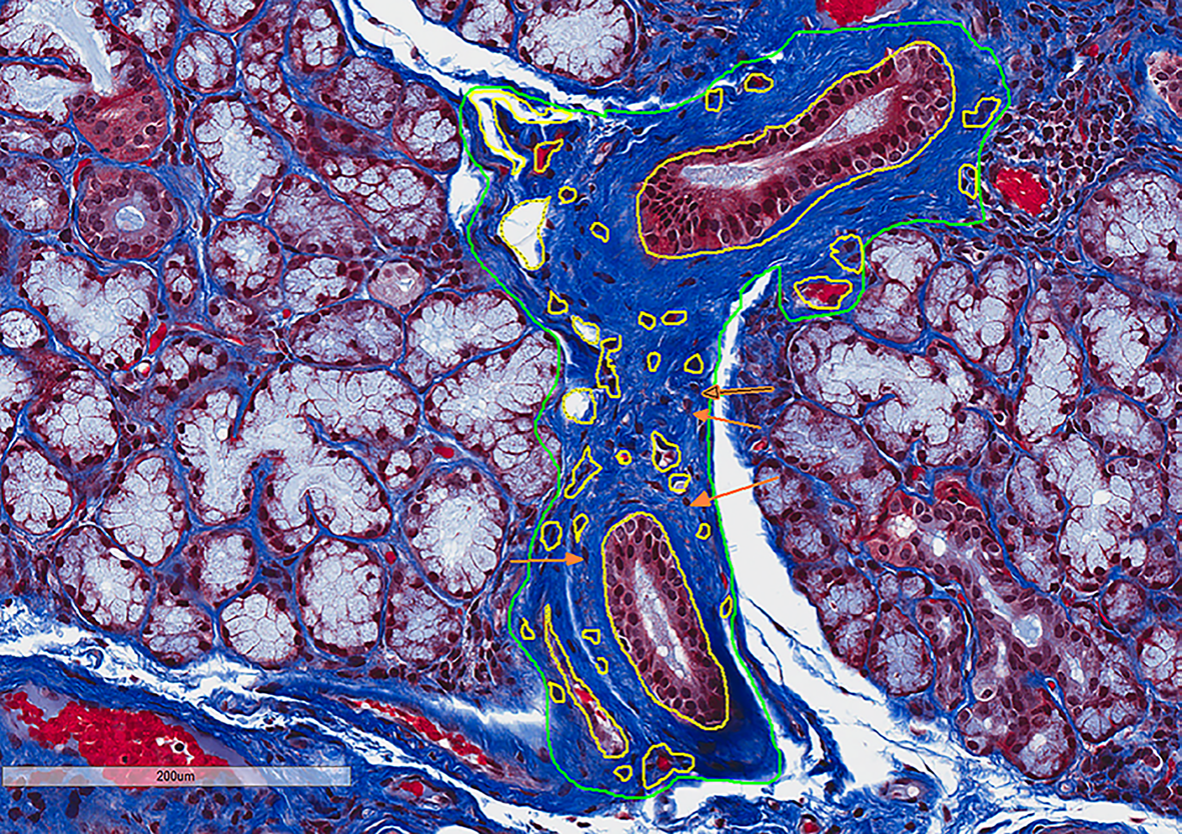
RIFA can be used to numerically quantify interstitial fibrosis in LSG from Sjögren’s syndrome patients. Masson’s trichrome staining was used to detect interstitial fibrosis in LSGs from Sjögren’s syndrome patients. Interstitial fibrosis areas and whole gland areas were measured using Aperio Scan Scope software. Non-fibrosis areas (outlined in yellow) that were included in the interstitial fibrosis areas (outlined in green) were subtracted to obtain accurate measurement. Area less than 50µm^2^ (orange arrows as example areas) were not excluded. RIFA was quantified as total interstitial fibrosis area (in mm^2^) per mm^2^ total glandular size on the section [(green circled areas – yellow circled areas)/total area].

To quantify the degree of interstitial fibrosis, we calculated the relative interstitial fibrosis area (RIFA) defined by interstitial fibrosis area (mm^2^) per mm^2^ total glandular size on the section using the equation shown below. The total interstitial fibrosis area was calculated by subtracting the size of any non-fibrosis area, if it was included in the fibrosis area, excluding non-fibrotic objects less than 50µm^2^ (for representative figure, see **Figure 2**):

RIFA=total interstitial fibrosis area†/total gland areatotal interstitial fibrosis area†=green circled areas−yellow circled areas

In addition, sample size calculations with a Student’s *t* test for unequal variances based on preliminary differences for RIFA in different microscopic groups showed that four samples would be sufficient to have at least 80% power to find differences with a 5% level of significance (https://www.stat.ubc.ca/~rollin/stats/ssize/n2.html). Therefore, a group size of five samples was considered a valid sample size to compare RIFA in different groups.

### Microarray Analysis to Identify Epithelial-Mesenchymal Transition–Associated Gene Expressions in Labial Salivary Glands of Sjögren’s Syndrome Patients

Epithelial-mesenchymal transition (EMT) long non-coding RNA/mRNA pathway microarray analysis was performed by Arraystar Inc. (Rockville, MD, USA) using LncPath™ Human EMT Pathway Microarray (Cat# AS-LP-004H). EMT coding genes were grouped and are listed in **Supplementary Table 2**. All genes that had already been proven to be associated with the EMT pathway were manually curated from the literature (22), and 219 potential coding targets related to the EMT signaling pathway were tested.

Total RNA was extracted from formalin-fixed, paraffin-embedded LSG biopsy samples from the 20 SS patients using an RNeasy Mini Kit according to the instructions of the manufacturer (Qiagen, Germantown, MD, USA). The RNA quality was measured using a 2100 Bioanalyzer (Agilent, Santa Clara, CA, USA). Only RNA samples with a 28S/18S ribosomal RNA ratio of 1.7 and an RNA integrity number of 6.5 were used for the arrays. Sample labeling and array hybridization were performed according to the manufacturer’s protocol (Arraystar Inc., Rockville, MD, USA).

Briefly, each RNA sample was amplified and transcribed into fluorescent cRNA along the entire length of the transcripts without 3’ bias utilizing a random priming method. The labeled cRNAs were purified using the same RNeasy Mini Kit (Qiagen). The concentration and specific activity of the labeled cRNAs (pmol Cy3/μg cRNA) were measured using NanoDrop ND-1000 (Waltham, MA, USA). One μg of each labeled cRNA was fragmented by adding 5 μl 10 × Blocking Agent and 1 μl of 25 × Fragmentation Buffer and kept at 60°C for 30 minutes, followed by the addition of 25 μl 2 × GE Hybridization Buffer to dilute the labeled cRNA. Fifty μl of hybridization solution was dispensed into the gasket slide and assembled to the microarray slide. Slides were incubated at 65°C in an Agilent Hybridization Oven for 17 hours, after which the hybridized arrays were washed, fixed, and scanned using the Agilent Scanner G2505C (Agilent, Santa Clara, CA, USA).

For statistical analysis of microarray data, quantile normalization of raw data and subsequent data processing were performed using the Limma package (R software, Bioconductor - limma). After normalization, low-intensity filtering was performed, and the coding genes with at least 4 out of 20 samples with a Present (P) or Marginal (M) signal flag were retained for further analysis. When comparing two groups, the “fold change” (i.e., the ratio of the group averages) between the groups for each coding gene was computed. The statistical significance of the difference was estimated using a Student’s *t* test. Genes with a fold change > 1.5 and a *P*-value < 0.05 were identified as significantly differentially expressed (DE)genes.

An online Venn diagram tool was used to identify common DE genes in group-group comparisons (SCS *vs.* NSCS or FLS/SCS) (http://bioinformatics.psb.ugent.be/webtools/Venn/).

### Protein-Binding Motif Matches in RIFA-Correlated Genes

Protein-binding motifs of transcription factors in the form of position frequency matrices were downloaded from the database CIS-BP (http://cisbp.ccbr.utoronto.ca). The program FIMO (Find Individual Motif Occurrences) from the Multiple Em for Motif Elicitation software suite [http://www.FIMO - MEME Suite (meme-suite.org)] was used to scan the human hg19 genome assembly for matches to these protein-binding sites. A “promoter” was defined as the 1000-bp upstream region of a canonical transcription start site. Matches to protein-binding motifs that overlapped with the promoter of a gene correlated with RIFA were collected. All transcription factors with a binding-site match were ranked by the number of RIFA-correlated gene promoters they overlapped with.

### Single-Cell RNA Sequencing Analysis of RIFA-Correlated Gene Expression in Salivary Gland Fibroblast Cells

Single-cell RNA sequencing (scRNA-seq) analysis was performed on fresh LSG cells from seven SS patients and five patients who did not meet the SS criteria (non-Sjögren’s subjects) (23). Data from these samples were analyzed in R (v3.5.0) using Seurat (v3.1.2). Filtering was performed using the standard quality control steps provided on the Satija Lab website (satijalab.org). Cells containing more than 200 and fewer than 2500 unique features were retained. From this set, cells with greater than 15% of their counts attributed to mitochondrial DNA were filtered out. We adjusted this value from 5% to 15% to increase the yield from each sample and did not observe substantial changes in our results upon making this adjustment. Data were normalized using the “NormalizeData” command (scale factor: 10,000).

LSG cells from all SS and non-Sjögren’s patients were integrated into a single Seurat object. When splitting this object into clusters based on cell RNA expression profiles, the resolution was set at 0.1 to identity nine unique clusters. The cell types corresponding to each cluster were identified with marker genes identified by Seurat’s “FindAllMarkers” function, using the receiver-operating-characteristic (ROC) analysis test. For clusters with an unclear identity based on the top 5 marker genes, the expression of genes known to be markers of specific cell types were visualized with Seurat’s “FeaturePlot” function. The localization of these transcripts was used to confirm the identity of any clusters in question.

Seurat’s “AverageExpression” function was used to determine the average expression of each gene of which the expression was significantly correlated with RIFA. To quantify each gene’s relative expression in fibroblasts and give each gene’s expression equal weight when averaging the expression of the list, gene expression values were converted to “percent of total expression” for each gene by dividing a gene’s expression in each cell type by the sum of its expression in all cell types. These results were visualized with Excel (v16.34; Microsoft, Redmond, Washington, USA).

### Promoter Accessibility in RIFA-Correlated Genes

Lung tissue was used as a surrogate tissue for assessing chromatin openness in place of salivary gland tissue due to the lack of data on salivary glands in public databases and because of its relatedness as a secretory tissue that undergoes branching morphogenesis. DNase I hypersensitive sites sequencing analysis (DNase-seq) reads collected from an upper lobe of a left lung sample from a 37-year-old male (ENCSR164WOF) were downloaded from the Encyclopedia of DNA Elements (www.encodeproject.org), as well as DNase-seq reads from a lung fibroblast sample from a 45-year-old male (ENCSR000EPR). The reads were aligned to the human reference genome hg19 using Bowtie 2 (http://www.Bowtie 2: fast and sensitive read alignment (sourceforge.net), and optical read duplicates were removed using Picard (http://www.Picard Tools - By Broad Institute).

A promoter was defined as a 1000-bp upstream region of a transcription start site, and overlapping promoters were merged into a single-promoter region. Per-base read coverage across the genome, as well as within genomic promoter regions, was estimated using bedtools genomecov and bedtools intersect (http://www.genomecov — bedtools 2.30.0 documentation) and averaged for each region. Coverage of promoters was compared between samples and gene sets using a two-tailed Student’s *t* test with Bonferroni correction.

### Statistical Analysis

Unpaired Student’s *t* test was used to compare the difference in RIFA or UWS flow rate between each two groups. Data are shown as mean ± standard error of the mean (SEM). Linear regression was used to analyze the correlation between RIFA and UWS flow rate or other clinical data. Pearson’s multiple variables correlation analysis was performed to identify the normalized gene expressions that significantly correlated with RIFA (*P* < 0.05) by using Excel (MS Office 365, Microsoft). All other analyses were performed with GraphPad Prism statistical software version 4.02 (GraphPad Software Inc., La Jolla, CA, USA). *P <*0.05 was considered to be statistically significant.

## Results

### Numerical Measurement of RIFA Is Comparable to Categorical Classification of Fibrosis in Labial Salivary Glands From Sjögren’s Syndrome Patients

To establish a numerical quantification of interstitial fibrosis, the RIFA was measured by assessing the interstitial fibrosis area (mm^2^) per mm^2^ of total glandular area on images from Masson’s trichrome–stained LSG sections (**Figure 2**). Five patients from each of the FLS, NSCS, FLS/SCS and SCS groups (20 in total) (**Supplementary Table 1**) were assessed.

**Figure 3:**
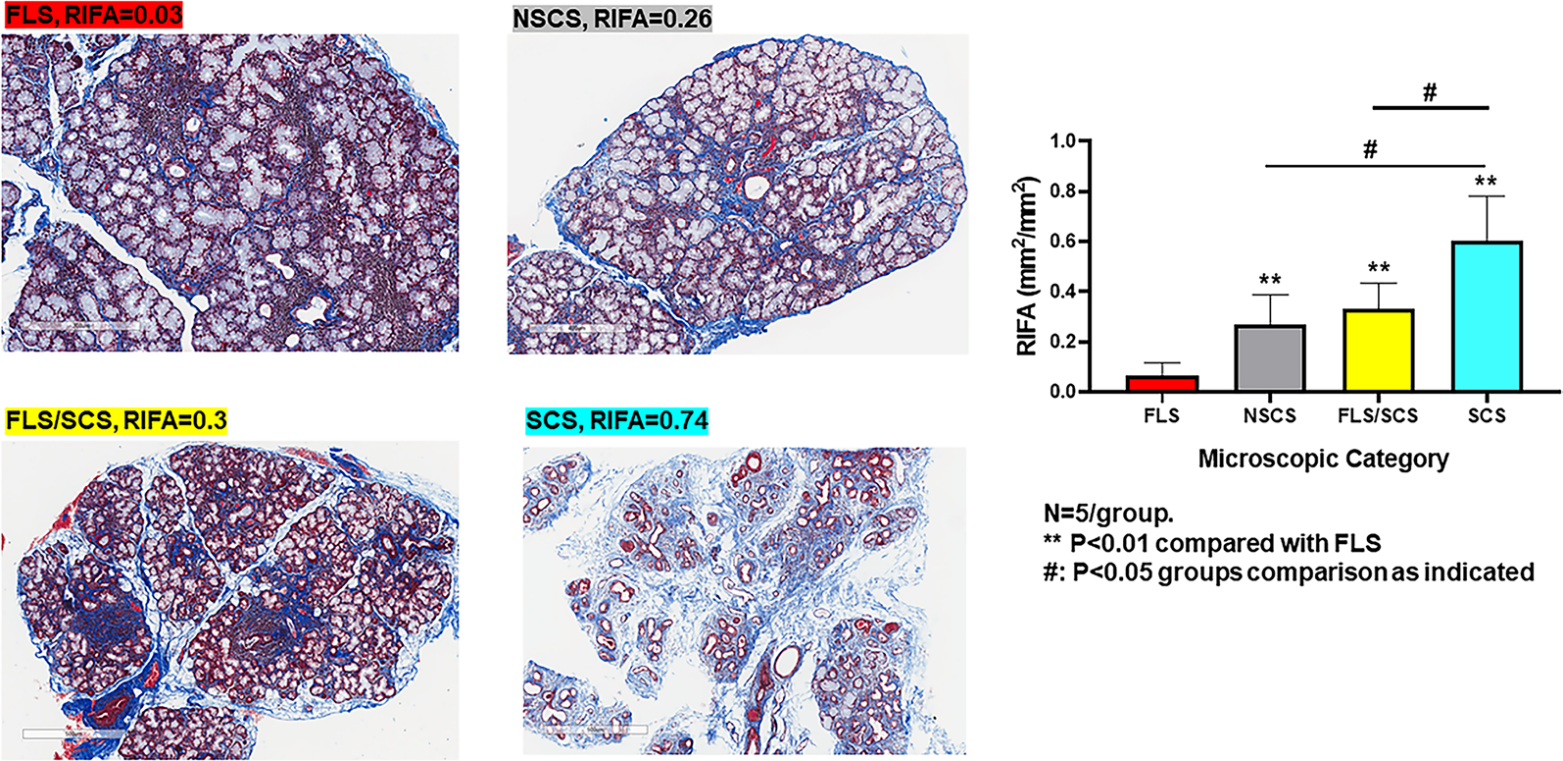
RIFA assessment corresponds with categorical classification of salivary gland interstitial fibrosis. Left: Representative images to detect interstitial fibrosis in the LSG from four different microscopic groups comprising FLS, NSCS, FLS/SCS and SCS. Right: Comparison of mean (±SEM) RIFA in four different microscopic groups: FLS, NSCS, FLS/SCS and SCS (n = 5/group). Statistical significance was determined with unpaired Student’s *t* test.

In the categorical classification defined by SICCA, patients with FLS have minimal or no fibrosis formation, whereas FLS/SCS and NSCS are characterized by intermediate fibrosis, and the most severe form of fibrosis is seen in SCS (4). RIFA measurements reflected this distinction in the classifications, as our SS patients with FLS, whose major microscopic change is lymphocytic infiltration, had the lowest RIFA score (0.06 ± 0.02; **Figure 3**). NSCS and FLS/SCS groups showed an intermediate level of interstitial fibrosis (0.27 ± 0.12 and 0.32 ± 0.10, respectively). Although there was no significant difference between these two groups, both had a significantly higher RIFA score than the FLS group (*P* < 0.01). The SCS group showed the highest RIFA score (0.60 ± 0.08), which was significantly increased compared with the NSCS, FLS/SCS and FLS groups (*P* < 0.01; **Figure 3**). These results suggest that as a numerical measurement, RIFA measurement is comparable to the microscopic classification methodology (4).

### RIFA Is Significantly Correlated With Salivary Gland Hypofunction in Sjögren’s Syndrome Patients

**Figure 4:**
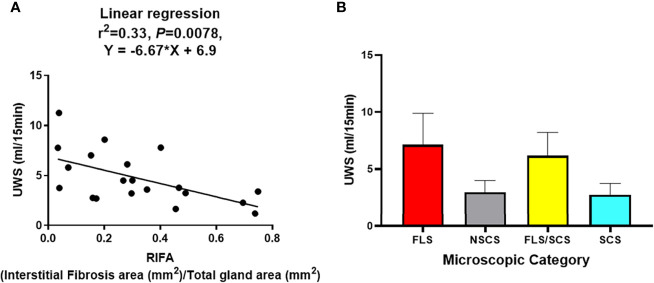
RIFA correlates with unstimulated whole saliva flow rate in Sjögren’s syndrome patients. **(A)** Correlation of RIFA and USW flow rate in Sjögren’s syndrome (SS) patients was analyzed using linear regression analysis. A significant negative correlation was found between RIFA and UWS flow rate (n = 20). **(B)** Comparison of mean (± SEM) UWS flow rate in different microscopic groups based on mean (± SEM) of RIFA measurement (FLS, NSCS, FLS/SCS, and SCS).

Linear regression analysis showed a significant negative correlation between RIFA and SS patients’ UWS flow rate (r^2^ = 0.33, slope = -0.67, *P* < 0.01; **Figure 4A**). However, no correlation could be found between UWS flow rate and the four categorical microscopic groups (FLS, NSCS, FLS/SCS, and SCS) (**Figure 4B**). In addition, no significant correlations between RIFA and other clinical data such as FS and autoantibody titer were identified (Pearson’s correlation analysis, *P* > 0.05, data not shown). This result indicates that the severity of salivary gland interstitial fibrosis is correlated with salivary gland hypofunction but not with other SS features.

### Transcriptome Analysis Using Microarray Data Identifies Gene Expression Profile That Is Associated With Interstitial Fibrosis in Sjögren’s Syndrome Patients

The above results support a link between fibrosis and the loss of gland function in SS. To identify the changes in gene expression associated with this link, microarray analysis was performed using RNA extracted from paraffin-embedded LSG biopsy samples from 20 SS patients (**Supplemental Tables 1, 2**). Correlation of RIFA with gene expression was determined by Pearson’s correlation analysis, and a list of 16 genes was found to be significantly and positively correlated with RIFA (*P* < 0.05). The correlation of RIFA with each of these 16 genes was further verified by linear regression analysis (**Table 1**, **Supplementary Figure 1**).

**Table 1:** Gene expressions significantly correlate with RIFA.

Gene Name	Description	Pearson’s r*
*SETD8* (*KMT5A*)	SET domain containing (lysine methyltransferase) 8	0.73
*NUMB*	numb homolog (Drosophila)	0.71
*BMP3*	bone morphogenetic protein 3	0.70
*PRKD1*	protein kinase D1	0.68
*TGFB2*	transforming growth factor, beta 2	0.66
*GSC*	goosecoid homeobox	0.65
*FZD7*	frizzled family receptor 7	0.65
*MT1F*	metallothionein 1F	0.58
*CXCL12*	chemokine (C-X-C motif) ligand 12	0.55
*ESR1*	estrogen receptor 1	0.54
*CDK4*	cyclin-dependent kinase 4	0.54
*MST1R*	macrophage-stimulating 1 receptor (c-met-related tyrosine kinase)	0.52
*FGFR2*	fibroblast growth factor receptor 2	0.52
*CDH1*	cadherin-1, type 1, E-cadherin (epithelial)	0.50
*CDH11*	cadherin-11, type 2, OB-cadherin (osteoblast)	0.49
*WNT4*	wingless-type MMTV integration site family, member 4	0.49

*P < 0.05 for all the listed genes (Pearson’s correlation analysis).

**Figure 5:**
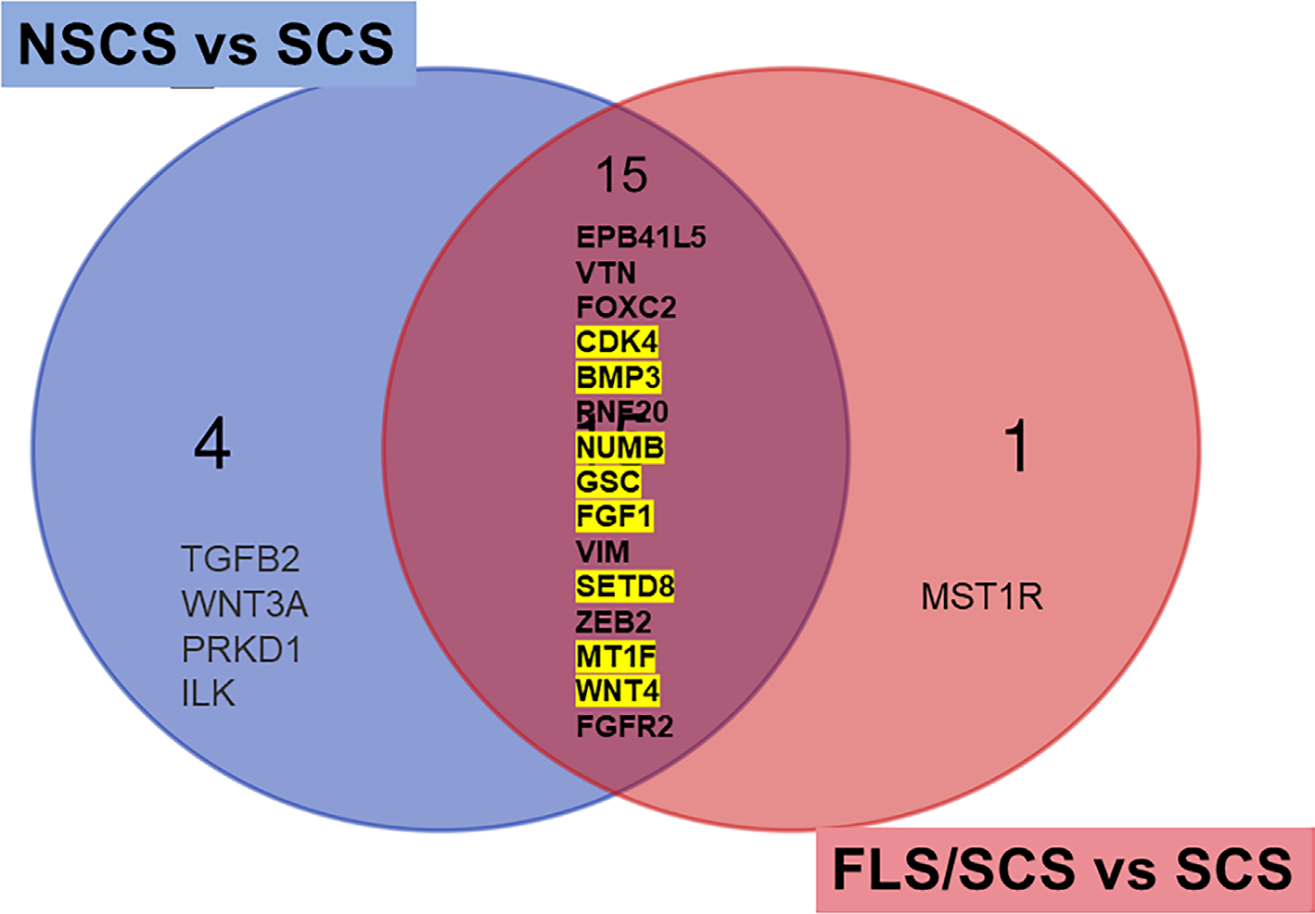
Differentially expressed genes associated with severe interstitial fibrosis are conserved among RIFA-correlated genes. Venn diagram to identify common differentially expressed genes in SCS group (severe fibrosis) compared with NSCS or FLS/SCS group (moderate fibrosis). Fifteen common upregulated differentially expressed genes from SCS group compared with moderate interstitial fibrosis NSCS group or FLS/SCS group were identified. Of these 15 genes, 8 genes were also significantly correlated with RIFA (highlighted in yellow, refer to **Table 1**).

Gene expression changes associated with severe fibrosis in SS were also investigated by comparing differentially expressed genes based on segregating samples into their SICCA defined microscopic groups. Upregulated differentially expressed genes in the SCS group (severe fibrosis) were compared with those in the NSCS group or FLS/SCS group (moderate fibrosis) (for heatmap, see **Supplementary Figure 2**). The common differentially expressed genes obtained by these two comparisons were likely associated with severe fibrosis formation (**Figure 5**). This list of differentially expressed genes included *CDK4*, *BMP3*, *NUMB*, *GSC*, *FGF1*, *SETD8* (*KMT5A*), *MT1F*, and *WNT4* (highlighted in yellow in **Figure 5**), which were also identified in the RIFA correlation analysis described above. The 50% conservation between these two lists suggests some consistency between RIFA measurement and SICCA’S microscopic classification for salivary gland fibrosis.

### Gene Expression Profile in Sclerosing Sialadenitis of Sjögren’s Syndrome Is Different From That in Idiopathic Pulmonary Fibrosis

Idiopathic pulmonary fibrosis (IPF) is a well-studied disease with lung interstitial fibrosis as the hallmark clinical and pathological change (24). Comparison of the sclerosing sialadenitis–associated fibrosis gene expression pattern identified in our study with published differentially expressed genes from the lungs of IPF patients (24) showed little overlap between salivary gland–associated fibrosis and IPF. Of all the transcriptome profile genes we identified as RIFA-correlated genes or as genes associated with severe fibrosis in SS, only the expression of *SETD8* and *CXCL12* was significantly upregulated in IPF patients compared with controls (**Table 2**). Therefore, while the pathological changes in the two diseases are similar, we found the transcriptome profiles for these two conditions to be distinct.

**Table 2:** Transcriptome profiling genes in current study compared with those in IPF study (24).

Fibrosis associated genes in this study	Gene expression changes in IPF study	
Gene Name	IPF *vs* Control	P<0.05
*EPB41L5*	↓	Yes
*VTN*		
*FOXC2*		
*CDK4*	↓	No
*BMP3*	↓	No
*RNF20*	↓	No
*NUMB*	↓	No
*GSC*		
*FGF1*	↓	No
*VIM*		
SETD8*	↑	Yes
*ZEB2*	↑	No
*MT1F*	↑	No
*WNT4*	↑	No
*FGFR2*	↓	No
*TGFB2*	↓	Yes
*WNT3A*	↓	Yes
*PRKD1*	↓	No
*ILK*	↓	Yes
*MST1R*	↑	No
*CDH1*		
*FZD7*	↓	No
*ESR1*	↑	No
CXCL12*	↑	Yes
*CDH11*	↓	Yes

*Common transcriptome profiling genes in both studies. ↑: Genes reported increased in IPF compared with control. ↓: Genes reported decreased in IPF compared with control.

### RIFA-Correlated Genes Are Regulated by Multiple Transcriptional Factors

Transcription factors act as master regulators in many cell state changes, such as EMT [for review, see (15, 16)]. To determine whether the RIFA-correlated genes are driven by a single “master regulator”, transcription factor motif analysis was used to identify transcription factors regulating the RIFA-correlated genes. Motifs for the transcription factor Kruppel-like factor 6 (*KLF6*) were found proximal to the start site of six RIFA-associated genes, namely *ESR1*, *FZD7*, *MST1R*, *CDK4*, *CDH1*, and *MT1F*. Other transcription factor binding sites were identified in fewer than five RIFA-correlated genes (**Supplementary Table 3**). This finding indicates that the RIFA-correlated genes are likely regulated by multiple transcriptional factors in SS.

### RIFA-Correlated Genes Expression Is Associated With Salivary Gland Fibroblasts

**Figure 6:**
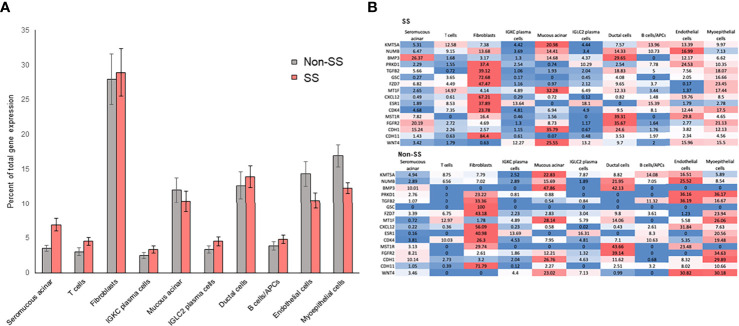
RIFA-correlated gene expression is primarily localized to fibroblasts in LSG from Sjögren’s syndrome and non-Sjögren’s patients. **(A)** Single-cell RNA sequencing analysis was used to quantify expression of RIFA-correlated genes in different cell types within LSGs from Sjögren’s syndrome patients and non-Sjögren’s subjects. Data shown are mean(± SEM) of “percent of total expression” of each gene within each cell type. Data revealed that RIFA-correlated genes were, on average, more highly expressed in fibroblasts than in any other cell type. **(B)** Assessment of “percent of total expression” of individual genes within each cell type revealed that eight genes were preferentially expressed by fibroblasts, which is reflected in increased average expression of list of genes in fibroblasts. Gene expression levels are represented on a color scale from high (red) to low (blue). The sum of values in a row equals 100% of the expression.

The above data suggest that multiple transcription factors are likely responsible for the expression of the 16 RIFA-correlated genes. Single-cell RNA sequencing analysis (scRNA-seq) was used to determine gene expression within specific cell types in LSG (**Supplementary Figures 3A, B**). The expression of RIFA-correlated genes was quantified in different cell types within LSGs from seven SS patients and five non-Sjögren’s patients to identify if a single cell type or multiple cell types contribute to the transcriptome profile. Averaging the “percent of total expression” of each gene within each cell type revealed that the average expression levels of RIFA-correlated genes were significantly higher in fibroblasts than those in other cell types (Bonferroni corrected *P* = 0.0179 and *P* = 0.0244 for SS and non-SS patients, respectively; **Figure 6A**). This effect appeared to be caused by a subset of RIFA-correlated genes, for which 23% to 84% of their total transcripts come from fibroblasts: *BMP3*, *PRKD1*, *TGFB2*, *GSC*, *FZD7*, *CXCL12*, *ESR1*, *CDK4*, and *CDH11* (**Figure 6B**). Many of the RIFA-correlated genes were more highly expressed per cell in the fibroblasts from Sjögren’s patients than in those from non-Sjögren’s subjects (e.g., *CXCL12*) (**Figures 6A, B**).

### Promoters of RIFA-Correlated Genes Are More Open in Lung Fibroblasts Than in Whole Lung Tissue

**Figure 7:**
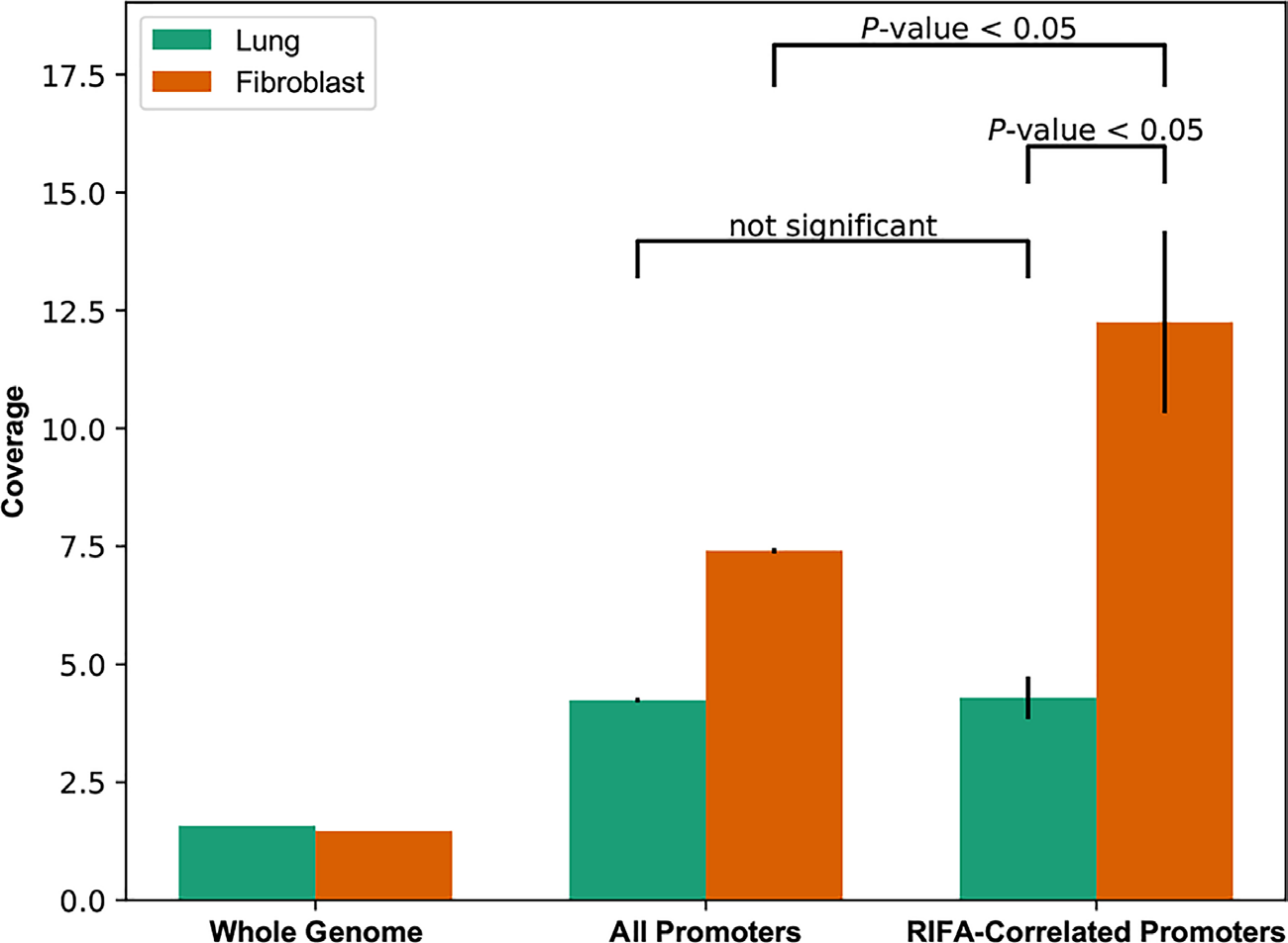
Core promoter openness is significantly higher for RIFA-correlated genes in lung fibroblasts than in whole lung tissue. DNase-seq reads were used to estimate chromatin accessibility of promoters of genes associated with fibrosis, all promoters, and the whole genome (as background) in both lung tissue and lung fibroblasts. Data shown are promoter coverage (mean ± SEM). Student’s *t* test was used to compare promoter coverage between pairs of groups.

The above results suggest that RIFA-correlated gene expression is mostly associated with the fibroblast population of cells in the LSGs. These data also indicate that the promoters are more likely “open” and accessible in these cells to allow gene expression. To determine the state of the promoters associated with these genes in interstitial fibrosis formation, accessibility of core promoter regions in fibroblasts derived from a lung sample was compared with those in whole lung tissue. In whole lung tissue, the openness of promoters of RIFA-correlated genes (promoter coverage: 4.71 ± 0.70) was not significantly different from that of all promoter regions across the genome (4.15 ± 0.02; *P* = 0.496). By contrast, in lung fibroblasts, promoters of RIFA-correlated genes were three times more accessible than the same promoters in whole lung tissue (12.94 ± 2.81 *vs.* 4.71 ± 0.70, respectively; Bonferroni-corrected *P* < 0.05), and they were significantly more open than the average for all promoter regions in fibroblasts (promoter coverage: 7.39 ± 0.06; *P* < 0.05; **Figure 7**). These data indicate that the RIFA-correlated genes are in a permissive chromatin state in fibroblast cells within epithelial tissues, which further supports the notion that these genes are involved in the fibroblast-driven sclerosing sialadenitis formation in SS.

## Discussion

Advances in personalized molecular pathology aim to build a multidimensional understanding of clinical manifestations, pathological changes, and molecular genetics, and this has led to the discovery of many candidate target therapeutics for diseases, such as cancer and autoimmune and fibrotic diseases (25, 26). Given the numerical nature of some clinical and molecular data, such as the gene expression matrix, development of a numeric variable to assess a pathological change will allow a better understanding of the associations between these dimensions using linear or non-linear correlation analysis (27–29). The emerging area of digital pathology provides more efficient and accurate assessment of individual microscopic changes in different diseases and can be broadly used to study the relationship between pathological change and clinical manifestations (26, 28, 29). For instance, in fibrotic diseases, such as osteosclerosis, numerical quantification with digital imaging correlates with subjective scores and provides accurate quantification of fibrosis (28).

Recently, Leehan et al. developed a digital measurement of salivary gland fibrosis, which has advanced the differential diagnosis of SS from non-Sjögren’s (11). In our study, we used Masson’s trichrome staining, a specific staining to identify fibrosis (collagen) in LSG biopsies. Using an image analysis program (Aperio Scan Scope) for digitally scanned slides, we directly quantified the interstitial fibrosis area without reconstruction of small grids. This limited the number of measurements of non-fibrosis area and improved precision. The assessment was approved by a board-certified oral pathologist.

Our results are in line with the previously published observations (11). Specifically, numerical quantification of fibrosis by RIFA allowed statistical analyses, such as linear regression and Pearson’s correlation analyses, to study the correlations of clinical data or gene expression changes with salivary gland interstitial fibrosis. Remarkably, we found that fibrosis assessment by RIFA measurement was significantly correlated with UWS flow rate in SS patients, suggesting a critical clinical pathological contribution of interstitial fibrosis in the development of salivary gland hypofunction. Furthermore, we identified genes that significantly correlated with RIFA, leading to a better understanding of the molecular changes involved in severe fibrosis formation in SS. Although our SS patient cohort was small, our findings may be important for the overall understanding of the pathogenesis and mechanism of xerostomia (subjective oral dryness) in SS.

Transcriptomic profiling of gene expression employing microarray analysis has been previously used to study the molecular changes in gene expression associated with fibrotic diseases such as IPF (24, 30, 31). Instead of reporting the differentially expressed genes in patients compared with healthy controls, we combined two approaches to assemble the transcriptome profile associated with salivary gland interstitial fibrosis in SS. For the first, we used Pearson’s correlation and linear regression analyses to identify RIFA-correlated genes. In the second approach, we compared gene expression patterns in a severe fibrosis group (SCS) with two intermediate fibrosis groups (NSCS and FLS/SCS).

EMT-associated gene expressions collaborate as a network and play a dominant role in the different stages of EMT progression and fibrosis formation [for review, see (16, 32)]. Of the 16 RIFA-correlated genes, *SETD8*/*KMT5A* can be regulated by *NUMB via* methylation. In addition, both *SETD8* and *NUMB* are involved in EMT-regulated diseases such as cancer (33, 34). However, neither of these genes has been broadly studied in the context of fibrotic diseases. We found both *SETD8* and *NUMB* were significantly correlated with RIFA (r is top ranked for Pearson’s correlation analysis, **Table 1**) and were also differentially expressed in the severe versus moderate fibrosis comparison analysis, suggesting that *SETD8* and *NUMB* are critical genes in salivary gland fibrosis development and severity in SS.

TGF-β and BMP signaling are two important inducers of EMT, which can be initiated by tissue inflammation [for review, see (16, 32, 35)]. TGF-β and BMP are known to be involved in fibrotic diseases in the lung, liver, and kidney and in systemic autoimmune diseases such as systemic sclerosis (36, 37). In SS patients, TGF-β is correlated with the inflammatory cytokine TNF-α (38). Koski et al. reported increased expression of TGF-β family member proteins, including TGF-β2, in the fibrous area of LSGs from SS patients, which were produced mainly by fibroblasts (39). The authors argued that TGF-β expression is initially increased as an immunoregulatory response to protect the focal epithelitis, which is followed by EMT induction to initiate a tissue healing. Conditional overexpression of TGF-β1 induces an SS-like phenotype with severe salivary gland fibrosis and xerostomia (40).

BMP6 is also associated with salivary gland hypofunction (19). In other fibrotic diseases, TGF-β family members are the major pro-fibrinous cytokines to initiate the EMT process and in turn promote fibrosis development, whereas BMP proteins often serve as antagonists of TGF-β. Interestingly, in our study, both *BMP3* and *TGFB2* expressions were increased in the severe fibrosis group and correlated positively with the degree of fibrosis in LSGs from SS patients. This difference in the TGF-β/BMP balance further suggests that sclerosing sialadenitis in SS patients has a molecular profile that is different from that of other fibrotic diseases.

*PRKD1* encodes protein kinase D1, a serine/threonine kinase that maintains the epithelial phenotype by preventing epithelial-to-mesenchymal transition (41). *PRKD1* is associated with fibrogenesis in mouse models and human kidney diseases, such as autosomal dominant polycystic kidney disease. Interestingly, *PRKD1* overexpression or mutation likely enhances TGF-β/SMAD signaling (42). *PRKD1* variants were reported in three families with mixed autoimmune disease including SS (43).

Although a direct relationship between SS or salivary gland fibrosis and FGF and the WNT families has not been reported, in our study, *WNT4*, fibroblast growth factor receptor 2 (*FGFR2*), and *FGF1* were significantly correlated with RIFA. *WNT3A* has also been identified as a differentially expressed gene in the severe fibrosis (SCS) versus moderate fibrosis (NSCS) comparison (**Figure 5**). Both these genes play a critical role during salivary gland morphogenesis and regeneration (44). TGF‐β1 can induce increased expression of FGF and FGFR2 in IPF, and ablation or inhibition of FGFR2 ameliorates lung and kidney fibrosis (45–47). Inhibition of FGFR disrupted the TGF‐β1-induced fibrosis process in a lung fibrosis model (45). The WNT family is able to regulate interstitial fibrosis formation by promoting β-catenin and driving the cells to produce collagen and α-smooth muscle actin to facilitate the EMT process (48, 49).

*CDH1* and *CDH11* expressions were significantly correlated with RIFA. CDH-11 is a main regulator and marker for mesenchymal cells that positively regulate EMT (50). The increased *CDH11* expression in our study suggests that activated mesenchymal cells play a critical role in salivary gland fibrosis formation in SS. Decreased expression of *CDH1*, which encodes cadherin-1, a critical marker for epithelial cells, is a hallmark change for EMT [for review, see (51)]. However, in our study, *CDH1* expression was significantly and positively correlated with RIFA, and it will require further study to understand its association with fibrosis in SS.

At the cellular level, EMT-related genes are reported to be overexpressed in many different types of cells, such as fibroblasts, epithelial, endothelial, and immune cells [for review, see (35, 51)]. Our scRNA-seq data showed that fibroblast cells are the primary source of RIFA-correlated gene expression in the salivary gland. Ductal, acinar epithelial, endothelial and myoepithelial cells also contributed to the RIFA-correlated gene overexpression, whereas immune cells, such as T, B and plasma cells, played a minor role in expression of RIFA-correlated genes. This suggests that although immune cells are the center of the autoimmune response, most EMT-related genes associated with salivary gland fibrosis formation and severity are mainly produced by cell types other than immune cells. It is likely that during the initiation of fibrosis, the interplay of fibroblast, myoepithelial, endothelial, ductal and acinar epithelial cells results in increased expression of specific genes, which leads to interstitial fibrosis formation.

**Figure 8:**
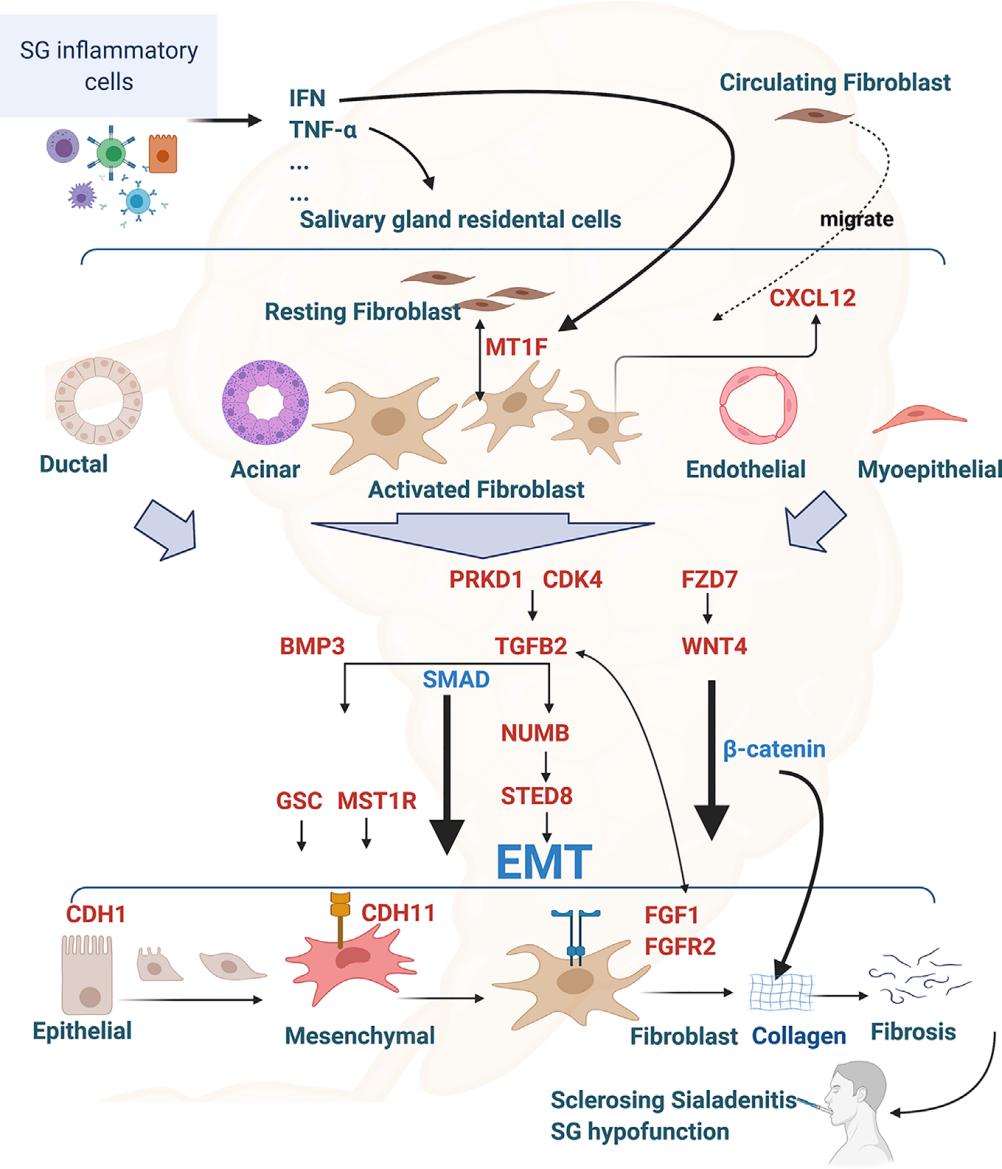
Interconnection of RIFA-correlated genes with epithelial-mesenchymal transition and salivary gland interstitial fibrosis formation in Sjögren’s syndrome. Illustration of the hypothesis of the EMT and interstitial fibrosis formation in Sjögren’s syndrome regulated by the16 RIFA-correlated genes. RIFA-correlated genes are identified in red. Figure is created with BioRender.com.

Based on the identified transcriptome changes associated with the scRNA-seq findings and the gene function reported in fibrotic diseases, we hypothesized that, following environmentally triggered inflammation, salivary gland immune cells produce pro-inflammatory cytokines associated with SS (e.g., TNF-α and interferon) that activate salivary gland epithelial, endothelial and myoepithelial cells and overexpress genes such as *TGFB2*, *WNT4*, and *BMP3*, which are key inducers of EMT. The salivary gland epithelial cells then transition to become mesenchymal cells, with markers such as *CDH11*, and ultimately transform to fibroblasts and produce collagen to form fibrosis during EMT. Increased *MT1F* expression can activate the proliferation of circulating fibrocytes [for review, see (16, 52)]. In response to *CXCL12* overexpression, activated fibroblast cells migrate to the inflammation-profibrotic area in the salivary gland (53). Meanwhile, through EMT initiation signals, such as TGF-β2, epithelial, endothelial and myoepithelial cells continuously overexpress EMT-promoting genes, such as *GSC* (54), *MST1R* (55), *SETD8* (*KMT5A*), *NUMB* (33, 34), *FGF1*, and *FGFR2* (45, 47), which in turn promotes fibroblast cell proliferation, migration, and transformation. As a positive feedback, the activated fibroblast cells overexpress *FZD7*, *PRKD1*, and *CDK4*, further activating *WNT4* and *TGFB2* expression (36, 56, 57). Collectively, the activated fibroblast cells overproduce collagens and extracellular matrix, resulting in interstitial fibrosis formation. Severe fibrosis and atrophy in the gland ultimately lead to salivary hypofunction in SS (for illustration, see **Figure 8**).

It is well known that epigenetic regulation through, for example, chromatin openness, contributes to the regulation of genes and is subsequently involved in various physiological and pathological changes (58, 59). Chromatin openness allows the interplay between chromatin-binding factors on promoters and reflects the readiness of associated genes for transcription. While global changes in chromatin accessibility have been reported in many other diseases, such as cancer and neurodegenerative diseases (18, 60), we found no significant difference in the chromatin accessibility at the whole-genome level between lung fibroblasts and normal human lung tissue (**Figure 8**). However, the chromatin openness of RIFA-correlated genes was remarkably higher in primary fibroblasts compared with normal lung tissue from an age- and sex-matched subject. This enhanced chromatin accessibility demonstrates the readiness of the RIFA-correlated genes to regulate salivary gland epithelial fibrosis formation. Through this analysis, we could detect epigenetic activation in the RIFA-correlated genes and concluded that these genes are highly important to fibroblast cells activation. Further epigenetic research on salivary gland interstitial fibrosis–associated genes will help us better understand the molecular mechanism associated with this clinical finding.

In conclusion, RIFA measurement is a promising method for assessing salivary gland interstitial fibrosis, and the quantification of the interstitial fibrosis should be evaluated as part of the pathological diagnosis of SS. The investigation of the molecular pathological analysis of salivary gland hypofunction will contribute to our understanding of the pathogenesis of sclerosing sialadenitis, and open a new avenue in personalized therapeutics for SS patients.

## Data Availability Statement

The datasets presented in this study can be found in online repositories. The names of the repository/repositories and accession number(s) can be found below: https://www.ncbi.nlm.nih.gov/geo/, GSE175709 https://www.ncbi.nlm.nih.gov/projects/gapprev/gap/cgi-bin/study.cgi?study_id=phs002446.v1.p1, 42170.

## Ethics Statement

The studies involving human participants were reviewed and approved by NIDCR, NIH. For single-cell RNA sequencing analysis, research subjects seen at BW’s Institute (NIDCR) reported herein provided informed consent before participation, according to NIH’s Single Institutional Review Board–approved research protocols (15-D-0051, NCT02327884). Except for the single-cell RNA sequencing analysis, all primary Sjögren’s syndrome (SS) patients’ data and specimens used in this study were obtained from the Sjögren’s International Collaborative Clinical Alliance (SICCA) Biorepository, funded under contract #HHSN26S201300057C by the NIDCR. This manuscript was prepared using a publicly available SICCA data set and does not necessarily reflect the opinions or views of the SICCA investigators, the NIH, or the NIDCR. The patients/participants provided their written informed consent to participate in this study.

## Author Contributions

HY, TP, BW, and JC participated in the conception and design of the experiments. HY, TP, BF, and NZ performed the experiments and data analysis. HY, TP, BF, BW, and JC wrote the manuscript, which was revised by all authors. All authors contributed to the article and approved the submitted version.

## Funding

The microarray analysis is supported by a National Institute of Dental and Craniofacial Research (NIDCR) grant to HY and JC. The single-cell RNA sequencing analysis was supported by funds to BW (NIDCR Z01-DE000704) through the National Institutes of Health (NIH) and National Institute on Deafness and Other Communication Disorders (NIDCD) Division of Intramural Research, NIH (ZIC DC000086). This work utilized computational resources from the NIH HPC Biowulf cluster. (http://hpc.nih.gov). The remainder of the research was supported by an NIH intramural grant to JC (1ZIADE000695).

## Conflict of Interest

The authors declare that the research was conducted in the absence of any commercial or financial relationships that could be construed as a potential conflict of interest.

## Publisher’s Note

All claims expressed in this article are solely those of the authors and do not necessarily represent those of their affiliated organizations, or those of the publisher, the editors and the reviewers. Any product that may be evaluated in this article, or claim that may be made by its manufacturer, is not guaranteed or endorsed by the publisher.

## Abbreviations

*SETD8 (KMT5A)*, SET domain containing (lysine methyltransferase) 8; *NUMB*, numb homolog (Drosophila); *BMP3*, bone morphogenetic protein 3 (encoded protein name: BMP3); *PRKD1*, protein kinase D1; *GSC*, goosecoid homeobox; *TGFB2*, transforming growth factor-beta 2 (encoded protein name: TGF-β2); *FZD7*, frizzled family receptor 7; *CXCL12*, chemokine (C-X-C motif) ligand 12; *MT1F*, metallothionein 1F; *ESR1*, estrogen receptor 1; *CDK4*, cyclin-dependent kinase 4; *MST1*R, macrophage-stimulating 1 receptor; *FGFR2*, fibroblast growth factor receptor 2; *CDH1*, cadherin 1, type 1, E-cadherin (encoded protein name: CDH-1); *CDH11*, cadherin 11, type 11, OB-cadherin (Osteoblast) (encoded protein name: CDH-11); *WNT4*, wingless-type MMTV integration site family, member 4; *WNT3A*, wingless-type MMTV integration site family, member 3A; *VTN*, vitronectin; *VIM*, vimentin; *ZEB2*, zinc finger E-box–binding homeobox 2; *FOXC2*, forkhead box protein C2.
