# Supplementary material for: Sclerosing Sialadenitis Is Associated With Salivary Gland Hypofunction and a Unique Gene Expression Profile in Sjögren’s Syndrome
Source: Front Immunol. 2021 Jul 30;12:699722. doi: 10.3389/fimmu.2021.699722 (PMC8363566; doi:10.3389/fimmu.2021.699722)
Supplement: Supplementary file 1 [file DataSheet_1.pdf]

Sclerosing sialadenitis is associated with salivary gland hypofunction and a unique gene expression profile in Sjögren's syndrome

Hongen Yin[1], Thomas J. F. Pranzatelli[1], Benjamin N. French [1], Nan Zhang[1], NIDCD/NIDCR Genomics and Computational Biology Core[2], Blake M. Warner[3], John A. Chiorini[1*]

[1] AAV Biology Section, National Institute of Dental and Craniofacial Research, National Institutes of Health, Bethesda, MD, United States

[2] Genomics and Computational Biology Core, National Institute on Deafness and Other Communication Disorders/ National Institute of Dental and Craniofacial Research, Bethesda, MD, United States

[3] Salivary Disorders Unit, National Institute of Dental and Craniofacial Research, National Institutes of Health, Bethesda, MD, United States

* Correspondence to: John A. Chiorini (jchiorini@dir.nidcr.nih.gov)

ABBREVIATIONS OF GENE NAMES

SETD8 (KMT5A): SET domain containing (lysine methyltransferase) 8

NUMB: numb homolog (Drosophila)

BMP3: bone morphogenetic protein 3 (encoded protein name: BMP3)

PRKD1: protein kinase D1

GSC: goosecoid homeobox

TGFB2: transforming growth factor-beta 2 (encoded protein name: TGF-β2)

FZD7: frizzled family receptor 7

CXCL12: chemokine (C-X-C motif) ligand 12

MT1F: metallothionein 1F

ESR1: estrogen receptor 1

CDK4: cyclin-dependent kinase 4

*MST1*R: macrophage-stimulating 1 receptor

FGFR2: fibroblast growth factor receptor 2

CDH1: cadherin 1, type 1, E-cadherin (encoded protein name: CDH-1)

CDH11: cadherin 11, type 11, OB-cadherin (Osteoblast) (encoded protein name: CDH-11)

WNT4: wingless-type MMTV integration site family, member 4

WNT3A: wingless-type MMTV integration site family, member 3A

VTN: vitronectin

VIM: vimentin

ZEB2: zinc finger E-box–binding homeobox 2

FOXC2: forkhead box protein C2

Supplementary Materials

Supplementary Table 1. Characteristics of the patients with Sjögren's Syndrome patients from different microscopic groups

	Microscopic groups			
	FLS	NSCS	FLS/SCS	SCS
Age ([years], mean, min-max)	55 (24-49)	47 (35-66)	40 (45-73)	65 (55-73)
Sex	Female	Female	Female	Female
Race (Caucasian/Hispanic/African American/Asian/Native American)	1/0/0/4/0	1/0/0/4/0	3/0/0/1/1	4/0/1/0/0
Anti-Ro/SSA positive	3	3	4	5
Anti-La/SSB positive	0	1	2	1
RF positive	4	4	4	1
ANA positive	3	4	2	2

Except where indicated otherwise, values are the number of subjects (N).

Supplementary Table 2 Microarray EMT Functional gene grouping

**Receptor Tyrosine Kinase:**
EGFR ERBB3 PDGFRB RGS2 SPARC
**Proliferation:**
CDK4 CCND1 CCND2 CCNG2 CDKN2A CDKN1A CDKN1B PCNA
**Alternative Splicing Regulators of EMT:**
CEBPD ESRP1 ESRP2 MBNL1 PTBP1 RBFOX2
**Motility and invasion:**
ACAN MMP13 MMP2 MMP3 MMP9 SPP1 RHOB CXCL12 SPARC VDR
**EMT Inducers:**
BMP1 BMP2 BMP3 BMP4 BMP5 BMP6 BMP7 EGF MMP28 FGF1 FGF2 HGF IGF1 IGF2 IL6 FAM3C JAG1 LAMB3 MMP13 MMP3 NRG1 NRG2 NRG3 NRG4 NOTCH1 NOTCH2 NOTCH3 NOTCH4 SPP1 FIGF PDGFA PDGFB PDGFC PDGFD VEGFB VEGFC POSTN SHH TGFB1 TGFB2 TGFB3 TMPRSS4 VEGFA VTN WNT1 WNT10A WNT2 WNT2B WNT3 WNT3A WNT4 WNT6 WNT7A WNT7B WNT8A
**EMT-inducing Transcriptional Factors (EMT-TFs):**

T ELSPBP1 TCF4 TCF3 ETS1 FOXC2 GSC KLF8 LEF1 SNAI1 SNAI2 TWIST1 ZEB1 ZEB2
**WNT:**
CTNNB1 FZD7 GSK3B WNT11 WNT5A WNT5B
**Epigenetic Regulators of EMT:**
BMI1 RNF20 SMARCA4 SMARCA2 OPN1LW DOT1L EZH2 EHMT2 HDAC1 HDAC2 HDAC3 HDAC4 HDAC6 KDM3A KDM4B KDM4C LOXL2 KDM1A MCM2 MCM3 MCM4 MCM5 MCM6 MCM7 NAP1L1 EP300 PKM RUVBL1 PRMT2 RUVBL2 SETD8 SIRT1 SUV39H1 SUZ12 TRIM33 WDR5
**Posttranslational regulation for E-cadherin:**
ARF6 MET NME1 SMAP1 SRC
**Posttranslational regulation for EMT-TFs:**
RASGRP1 CSNK1A1 CSN2 FBXL14 PCBP1 LATS2 SLC39A6 MDM2 PAK1 PRKD1 YBX1
**Epithelial Markers:**
DSP JUP CDH1 LAMA1 MUC1 TJP1
**Targets subject to EMT-associated alternative splicing:**
ARHGAP17 ARHGEF11 BAIAP2 CD44 CTNND1 DNM2 ENAH EPB41L5 FGFR2 FLNB ITGA6 LRRFIP2 MAGI1 MAP3K7 NUMB RALGPS2 MST1R SCRIB SLK TCF7L2
**Estrogen Receptor:**
CAV2 ESR1 KRT19 TGFB3
**Translational Regulators of EMT:**
PCBP1 YBX1
**G-Protein Coupled Receptor:**
AKT1 FZD7 GNG11 RAC1 RGS2
**Notch:**
FOXC2 JAG1 NOTCH1
**TGF/BMP:**
BMP1 BMP2 BMP7 COL3A1 SMAD2 TGFB1 TGFB2 TGFB3
**Survival:**
ATM BCL2L1 BID CXCL1 DFFB FOS FOXG1 MST4 MT1A MT1B MT1E MT1F MT1G MT1H MT1M MT1X TP53 TP73 BBC3
**Mesenchymal Markers:**
DDR2 FN1 S100A4 LAMA3 CDH12 CDH2 CDH11 SDC1 VIM CTNNB1
**Integrin-Mediated:**
COL3A1 ILK ITGA5 ITGAV ITGB1 PTK2

Supplementary Table 3 Transcriptional factors related to the 16 genes significantly correlated to RIFA

Transcription Factor	Total Promoters Present	Regulated RIFA correlated genes
KLF6	6	*ESR1,FZD7,MST1R,CDK4,CDH1,MT1F*
MECP2	4	*CDH1,MT1F,BMP3,FZD7*
FOXM1	3	*CDH1,BMP3,ESR1*
FOXD3	3	*CDH1,TGFB2,BMP3*
FOXF1	3	*CDH1,TGFB2,BMP3*
INSM1	3	*TGFB2,CDK4,ESR1*
SP4	3	*MT1F,MST1R,FZD7*
NR0B1	3	*BMP3,CDK4,SETD8*
FOXJ2	3	*CDH1,TGFB2,BMP3*
RUNX3	3	*MT1F,BMP3,ESR1*
ZNF350	3	*SETD8,MT1F,FZD7*
MBD2	3	*SETD8,MT1F,BMP3*
SP1	3	*MT1F,MST1R,FZD7*
EGR4	3	*SETD8,MT1F,MST1R*

Supplementary Figures and Legends

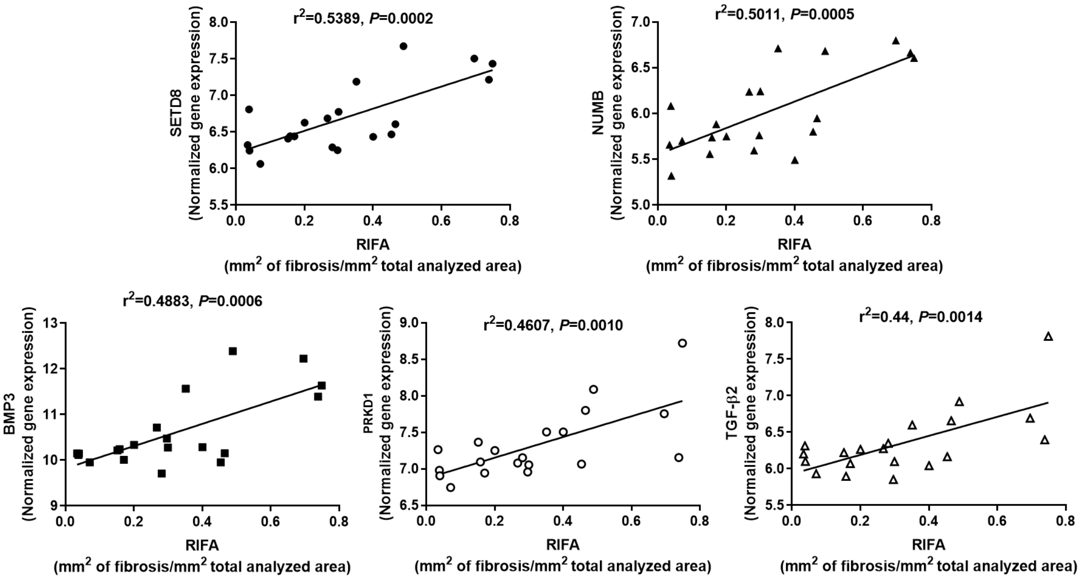

Supplementary Figure 1. Top 5 genes with expression that significantly and positively correlates to RIFA in Sjögren's patients. The correlation between RIFA and gene expression was analyzed using Pearson analysis. Sixteen genes were found to significantly and positively correlate with RIFA (Table 1). Linear regression was used to confirm the correlation of the top 5 genes based on Pearson's P value rank (N=20, R^2 and P value are as indicated).

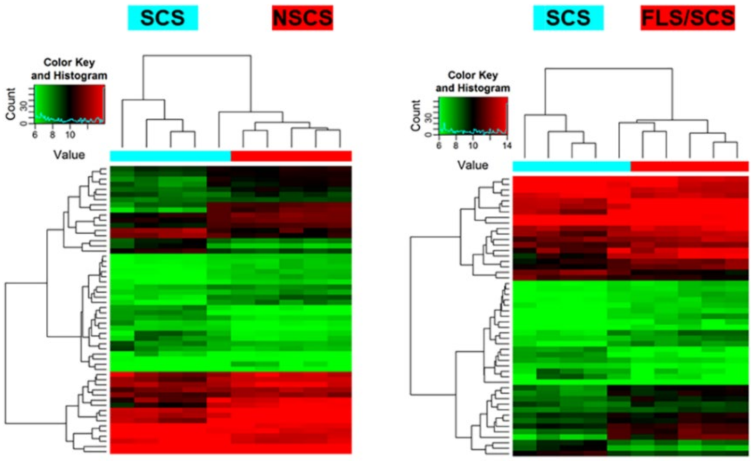

Supplementary Figure 2. Differentially expressed (DE) genes in SCS group compared with NSCS or FLS/SCS group. Microarray EMT gene expression cluster was shown as heatmap for SCS group compared with NSCS (left) or FLS/SCS (right) group.

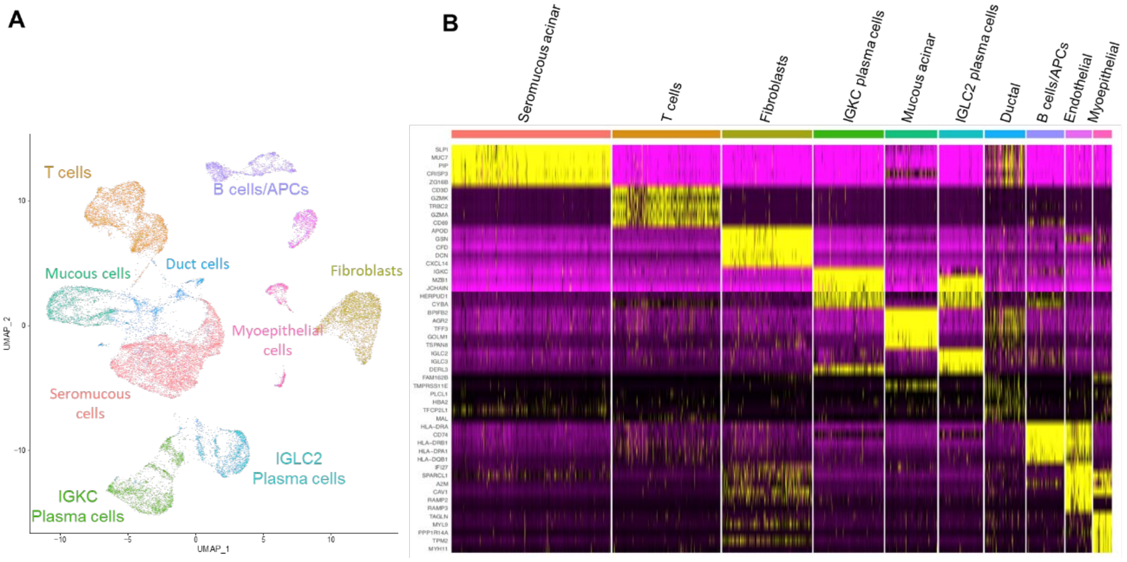

Supplementary Figure 3. Gene expression within specific cell types in salivary glands from Sjögren's and non-Sjögren's patients. Single cell RNA-seq (scRNA-seq) was used to understand gene expression within specific cell types in salivary glands from Sjögren's and non-Sjögren's patients. **A)** UMAP of different cell types in salivary glands; **B)** Computationally determined maker genes and FeaturePlots of select expression of canonical cell types with which

cell identities were determined. Bright yellow coloring corresponds to a high expression Z-score and bright pink coloring corresponds to a low expression Z-score.
